# Reports of Cases Occurring in the Medical Ward of the Buffalo Hospital

**Published:** 1858-03

**Authors:** A. W. Nichols

**Affiliations:** Assistant to Chair of Clinical Medicine, University of Buffalo


ART. IV.—*Abstract of the Clinical Lectures delivered at the Buffalo Hospital of the Sisters of Charity, upon the Diseases treated in the Male Medical Ward, by* AUSTIN FLINT, M. D., *Attending Physician, during the present College Session, viz., from October 14th,* 1857, *to February* 27, 1858. Reported by A. W. NICHOLS, M. D., Assistant to Chair of Clinical Medicine, University of Buffalo.

No. IV. *For January.*

*Paralysis; Hemiplegia; Paraplegia; Modifications of Sensibility; State of the Mind.*

Paralysis may be defined to be abolition or diminution of motion in some part or parts of the body, usually accompanied with total loss or diminution of sensation.

There are several varieties of paralysis, too well known to claim description, such as hemiplegia and paraplegia. There may exist total loss of motion in the affected parts; but more frequently some degree of motion remains , forming what is called *partial* paralysis. When limited to a particular region of the body, it takes the name of *local* paralysis. Where sensation remains and motion is impaired or lost, the paralysis is *incomplete.*

The causes of this affection reside in the nervous system, either in the brain or in the spinal cord, or as in local palsy in the nervous trunks proceeding thereto. Paralysis is not strictly a disease *per se,* but a sequel or consequence of some morbid process. When a hemisphere of the brain is the seat of disease producing paralysis, hemiplegia is the result; this may be more or less extensive, according to the amount of injury the brain-substance has received. Paraplegia, on the other hand, is due to some disease or lesion of the spinal cord. Whilst local palsy, as just remarked, usually depends on some local cause interfering with the function of the nerve or nerves distributed to the part.

The diseases which generally occasion hemiplegia, are apoplexy, accompanied with hæmorrhage, and softening of some portion of the brain. In hemiplegia, the symptoms characterizing the affection, may not become known immediately, particularly if it succeed an attack of apoplexy. When the effects of compression due to the amount of hæmorrhage, have passed away, and the patient regains his consciousness, the loss of voluntary movement over more or less of one side of the body is rendered apparent.

It is interesting to observe the symptoms produced by a considerable amount of hæmorrhage into the brain-substance like that which commonly takes place in apoplexy. We may state, very briefly, that the effused blood produces compression of the brain; this is shown by the comatose condition of the patient after such an attack. The hæmorrhage having ceased, the serum of the blood is absorbed, leaving behind the clot. The cause of compression being thereby almost removed, coma disappears, and the individual regains his consciousness to a greater or less degree; and paralysis is discovered to exist over more or less of one side of the body. But the clot of blood which remains in the substance of the brain, gives rise to a series of events very similar to those produced by any foreign body; although not precisely the same. It should be borne in mind that the clot may be almost entirely absorbed. And further, that its constituents are similar to those which exist in the neighboring structure. The clot then excites a certain amount of inflammation in the substance of the brain immediately surrounding it; and in this way, we account for that change which takes place in the condition of the patient, manifested by active delirium. The disease to all appearances, progresses favorably for a few days, when the patient is attacked with delirium manifested by incoherent talking, repeated attempts to get out of bed, accompanied with a flushed face and animated or excited countenance; the pulse, instead of being slow and feeble, becomes frequent, quick, and full; and the general aspect of the patient contrasts strongly with his recent lethargic or semi-conscious condition. After a time, these symptoms disappear according as the inflammation subsides.

It is unnecessary, however, for our present purpose, to pursue this interesting subject any farther. The patient having passed through this stage of the disease, slowly and partially regains his strength; and long after he is able to move the unaffected members of the body so as to rise from the bed, does he make the attempt. This disinclination to get out of bed, is a singular feature of many cases of hemiplegia, and doubtless may be due to the weakened condition of the mind which we are about to notice.

Hemiplegia does not always depend on a prior apoplectic attack for its production. It may be due to a tumor within the skull; or more frequently to softening of the brain. When it proceeds from these latter causes, it comes on slowly almost imperceptibly. This was well illustrated in the case of a patient now under treatment. He had been in bed several weeks, complaining of debility and of difficulty in moving the lower extremities sufficiently to enable him to rise from the bed. He then began to experience a sensation of coldness or numbness in both upper and lower extremities of the right side only; and on examination was found to be paralyzed on that side of the body. As there was no evidence of any cerebral hæmorrhage in this case, the cause of the paralysis may probably be attributed to softening of the brain.

Paraplegia originating as it does from some injury to or disease of the spinal cord, does not involve, in the majority of cases, any disturbance of the great nervous centres. The patient sometimes being attacked suddenly, as occurred in one case from prolonged bathing in cold water, and in another case from a fall which fractured the humerus; and sometimes slowly as was the case in a patient under treatment some time since.

Aside from the loss of voluntary motion in the affected limbs, sensation may be changed in various modes. It is no uncommon event for the individual suffering from paralysis, to complain of distressing pains in the affected members- And still less infrequent is it to meet with cases of paralysis in which sensation is very materially impaired.

In persons of the latter class, various experiments may be made successively on the healthy and on the paralyzed extremities, to ascertain the amount of sensibility remaining. When the appreciation of pain is entirely wanting, the term *analgesia* has been applied to this condition.

But, besides alterations in sensation or in the cognizance of painful impressions in the affected part, another interesting feature is occasionally observed, namely, loss of *tactile sensibility*, or the common sensation of touch which has been regarded as one of the five senses. To this state, the term *anæsthesia* may properly be applied.

In cases of paralysis, we may then meet with those which present a diminution or loss of painful impressions or of sensation, called analgesia; the tactile sensibility remaining unimpaired. And with others in which there exists a loss of the sensibility to touch, whilst the capability of receiving painful impressions is very little, if at all impaired; these latter forming instances of anæsthesia.

To illustrate these conditions of sensation in paralysis, there are now three patients in the ward: two are affected with hemiplegia, and one with paraplegia. In both the former cases, the appreciation of painful impressions is much diminished; this is shown by piercing the skin with a pin, and carrying it to some depth before pain is experienced. But in both these patients the tactile sensibility is preserved; this is rendered evident by their ability to walk quite well with their eyes closed or their attention withdrawn. They are conscious when they touch the floor with the affected foot, or when they grasp any object with the affected hand; farther, they appreciate the impression made by gently touching the paralyzed parts, but are conscious of no pain when the same parts are severely pinched.

A French observer, M. Beau, was the first to point out these peculiaritie of sensation, and deduced conclusions in favor of the opinion that there existed throughout the surface of the body, a special set of nerves constituting the sense of touch, just as is the case with the senses of smell, sight, hearing, and tasting. Farther observations in reference to these phenomena are desirable in order to decide upon the accuracy of his views.

A very common impression has prevailed in the minds of physicians, that hemiplegic patients, after the immediate effects of the apoplectic attack have passed away, do not usually regain their former mental condition. In other words, they often become imbecile or deficient in intellect. Peculiar circumstances recently occurred in the city of New York, directing the attention of the profession to this subject; and one of the physicians to Bellevue Hospital, Dr. Benj. W. McCready, has written a very elaborate article on the condition of the mental faculties after an attack of apoplexy followed by hemiplegia. This paper was published in the New York Journal of Medicine, Sept., 1857.

Dr. McC. draws his inferences respecting the state of the mind after the occurrence of these events, from collections of cases of apoplexy already reported. His deductions are, therefore, free from any preconceived opinions or from any tendency in favor of his views in the report of individual cases.

The result of his investigations goes to show, that the common impression of the profession is, in a great measure, incorrect. That the mental condition of those who have suffered from apoplexy and hemiplegia, is not very materially different from what it was prior to the attack. In Mr. Copeman's collection of cases of apoplexy, there are fifty cases, "in which the patient had recovered from the first effects produced by the apoplectic stroke;" twenty-four of the fifty cases, were followed by hemiplegia. Yet, "in no one of these cases," says Dr. McC., is "there the slightest indication that the patient was left with a mind, I will not say, reduced to a state of imbecility, but impaired in any marked degree."

Proceeding to higher authority, he then analyzes the cases contained in the *Clinique Médicale* of Andral, and in the *Anatomie Pathologique* of Cruveillier. They are sixteen in number, in which there existed hemiplegia due to an attack of apoplexy; these patients subsequently died of other diseases. But, in only two instances was there noticed any decided impairment of the mind. In many individuals, the subjects of the foregoing analyses, their customary avocations were resumed, and their mental faculties as freely and as severely exercised as antecedent to the apoplectic seizure.

The symptoms which are presented during the course of the disease, after the comatose state has disappeared, aud hemiplegia has been discovered, are such as to induce the friends or attendants to believe the patient imbecile, or even idiotic. And consequently, the hemiplegic has not unfrequently been consigned to the care of nature alone. And it is to be feared that the isolation and neglect to which these unfortunate persons are often subjected, has actually been productive of much of the imbecility or idiocy occasionally witnessed in such cases. "The mind rusts out for want of exercise!" The phenomena referrible to the faculties of the mind are well worthy the study of the physician. Dr. M'C. remarks upon this subject, that apoplectics, "comprehend well and judge correctly, but before their general health is confirmed, they can no more think continuously than they can take a long walk, or perform any other act demanding a considerable expenditure of nervous force." The memory, he adds, is "most apt to be impaired." In other cases, the faculty of speech is impaired or lost: the memory of words also may be lost, or words may be used improperly. In this paper, cases are adduced in which these phenomena occurred, and where the mind was not affected.

The following cases of hemiplegia were recorded prior to the discussion of this subject. They were under treatment in the medical ward of the Hospital for a considerable period of time: sufficiently long for any impairment of the mental faculties to have been recognized.

Case I. *Apoplexy; Right Hemiplegia; Loss of Memory of Words; Lately, Epileptiform Convulsions.*

April 17th, 1856: Patrick Doherty, Irish, aged 40, laborer; admitted yesterday. No definite account of his previous history could be obtained, except that he had been affected with paralysis of the right side of the body.

He lies with his eyes open, notices what is going on arcund him, and his countenance does not denote a lack of intelligence. To all questions addressed, to him he invariably answers, "yes." This is the only word he speaks at any time. He even asks for nothing in the way of food or drink. But, when these are brought to him, he partakes of them. He makes a partially successful effort to protrude the tongue, when directed to do so. He makes motions for the attendants to furnish him with the vessel, and he uses it for his evacuations.

It is a matter of doubt, at this time, whether he is in a state of mental imbecility or not.

May 25th. Patient remains in about the same condition: he has improved in strength, however.

He never speaks except when questions are addressed to him, and then he uniformly answers, "yes." His countenance does not show any evidence of deficiency in intellect.

August 1st. Patient is able to walk with a little aid. He has scarcely any power over the right upper and lower extremities. When asked to protrude the tongue, he makes ineffectual efforts to do so. It is a difficult matter to determine how far the inability to talk proceeds from imbecility or from want of power over the muscles of the tongue and larynx. He still says nothing, except when questions are put to him: then he answers by the word, "yes." When the question demands a negative answer, he replies, "yes," and shakes his head. He evidently comprehends to a certain extent, the import of questions. And his aspect does not denote any impairment of intellect.

October 14th, 1857. This patient has been in the hospital since last record. He remained several weeks. He presented the same symptoms and the same mental condition as at last record. He entered the hospital again this fall; and now appears to be in much the same state of body and mind as when seen last, in April, 1857.

He is troubled with diarrhœa, and comes in to be treated for this. His aspect is bright. His countenance does not show any want of intelligence. He never speaks except when questions are put to him, and his only answer is, "yes." He comprehends the meaning of what is said to him. He beckons for food and drink, and for his other wants. He recognizes former friends in the hospital. He knows his wife, who comes at the interval of a week or more, to visit htm. He attempts to speak, and makes ineffectual efforts to protrude thc tongue, when directed. The amount of intelligence which he exhibits is as much as at any period since he has been under observation: he might be pronounced more intelligent. Nothing of interest occurred in this case until he left the hospital on the 30th of October, 1857, except, that during the month he had several convulsions, resembling an attack of epilepsy, by which he was very much prostrated. But he left the hospital very much in the same condition of body and mind as when observed on the 14th of the month.

Case II. *Previous Apopletic Attack doubtful; Left Hemiplegia; Partial Improvement; State of Mind twenty-two months after attack.*

May 20th, 1856. Ed. McGowan, Irish, aged about 54, laborer: admitted last evening. The occurrence of apoplexy can not be ascertained, but is probable. He was suddenly attacked with paralysis of left side, on the 16th inst.

He was in a semi-comatose condition. He could be roused with some difficulty; and then he replies to questions, and makes efforts to move the limbs.

His mind is not active; but he tells his age; how long he has been sick, &c., &c.

He complained of pain in the right side of the head.

The sensibility appeared to be unimpaired.

From the 22d of May to the 6th of June, the patient exhibited the symptoms of that condition which have been supposed to be due to the inflammation excited by the presence of the clot in the brain, and which have been briefly adverted to above.

July 15th. This patient has improved very materially. He now sits up, and is able to walk a little. His general health is excellent. His mind is quite bright. He is full of Irish humor; and makes the ward merry with his jokes.

September 25th. Has acquired considerable power in the paralyzed limbs. Can walk out every day, and to some distance from the hospital.

He appears bright in intellect, and his mind is quite active. His humorous conversation is far above that expected in an individual in his condition of life.

February, 1858. This patient has been in the hospital since date of last record. He has been employed about the hospital; and has occasionally applied for relief for some transient symptom.

His mind is clear and active. He converses freely and intelligibly. His mental faculties are good. During a portion of the summer, he has occupied his time in the instruction of the little children in the hospital.

Case III. *Apoplexy; Left Hemiplegia; State of Mind two months after attack; Death by Asthenia.*

July 11th, 1856. M. B., American, aged 43, miner: admitted on the 9th inst. Was attacked with apoplexy on the 7th inst. There existed paralysis of motion on the left side. Sensibility is not lost; but he experiences in the affected limbs the feeling of increase in bulk.

Passing over the numerous symptoms connected with the hemiplegia, we come to the state of the mind. His intellect is clear. He replies to questions intelligibly; but he manifests some forgetfulness of the circumstances attending his recent ataack.

His knowledge of prior events appears to be excellent. He asks for what he desires, and converses intelligently.

He suffers very much from pain in the affected limbs.

From July 17th to August 9th, the patient exhibited those symptoms which are supposed to indicate inflammation of the brain substance surrounding the clot.

August 31st. Patient has exhibited a great disinclination to get up. He offered no resistance when the attendants proposed to assist him. And he has been up for a short time during the past two weeks.

September 17th. His mind is clear. He converses with those about him intelligibly. He makes known his wants to the attendants. He answers questions correctly. He is very weak, however; and instead of growing stronger, has become much weaker.

He died by asthenia at half-past ten P. M. Was conscious an hour before decease.

In regard to the treatment of these patients, it will be unnecessary to add any thing to the measures commonly adopted during the state immediately subsequent to the apoplectic attack.

If the patient survive the effects of the apoplexy itself, the helpless condition in which he is left by the paralysis of more or less of one side of the body, calls for our greatest care, and our most watchful attention. Hitherto, perhaps, we have regarded too exclusively the loss of physical power, and have directed most, if not all, our measures to the relief of the paralysis. But with the present knowledge of the mental condition of these truly unfortunate persons, much doubtless may be done to engage their attention, to call forth their mental faculties, and to exercise these gently at first; but sooner or later, to restore the mind to such an extent, that the person may be of much usefulness, if not really to regain his former strength of mind. At any rate, by this plan, we will not contribute to the isolation and neglect which such cases sometimes, perchance frequently, receive; and thereby avoid as far as lies in our power, the tendency to imbecility or idiocy, which is doubtless owing—in not a few instances—to the want of any exercise of the mind. Like as in a paralyzed limb, the muscles, for want of exercise, waste away, or become the seat of fatty substitution; so in the hemiplegic person, the brain, for a similar reason, may become atrophied or softened, or the seat of the same substitution.

